# Lymphangioma of the Oral and Maxillofacial Region: A Report of Three Cases

**DOI:** 10.7759/cureus.32577

**Published:** 2022-12-15

**Authors:** Naqoosh Haidry, Peeyush Shivhare, Aiswarya Vaidyanathan, Ejaz Ahmad Mokhtar, Aayushma Chapagain Ghimire

**Affiliations:** 1 Oral and Maxillofacial Surgery, All India Institute of Medical Sciences, Patna, Patna, IND; 2 Oral Medicine and Radiology, All India Institute of Medical Sciences, Patna, Patna, IND; 3 Dentistry, Nobel Medical College Teaching Hospital, Biratnagar, NPL

**Keywords:** bleomycin, sodium tetradecyl sulfate, sclerotherapy, lymphatic malformations, lymphangiomas

## Abstract

Lymphangiomas, or lymphatic malformations (LM), are benign malformations of the lymphatic system characterized by abnormal proliferation of lymphatic vessels. It was first described by Virchow in 1854. They occur rarely in the oral cavity and involve the tongue dorsum more often. Though complete surgical excision is the gold standard and most desirable management, certain limitations restrict this approach. Laser therapy, cryotherapy, electrocautery, sclerotherapy, and intralesional injections of steroids and Bevacizumab are other treatment options in such cases. Here, we present three cases of diverse forms of lymphatic malformations treated with two different modalities of treatment.

## Introduction

Vascular anomalies or lesions are congenital, localized structural defects of the vascular system involving arteries, veins, or lymphatics [1]. The most recent classification accepted by the International Society for the Study of Vascular Anomalies 2018 (ISSVA) classifies vascular anomalies into vascular tumors and vascular malformations. Vascular malformations can be subdivided into capillary malformations, lymphatic malformations (LM), venous malformations, arteriovenous malformations, and arteriovenous fistulas [2].

Lymphangiomas, or LM, are benign malformations of the lymphatic system characterized by abnormal proliferation of lymphatic vessels [3]. Blockage of the lymphatic system during fetal development results in congenital lymphangiomas, while acquired lymphangiomas can be due to trauma, inflammation, malignancy, surgery, radiation therapy, and lymphatic obstruction. Cystic lymphangiomas can be linked to different syndromes, such as Noonan syndrome, Turner syndrome (trisomies 13, 18, and 21), and Down syndrome [4].

Various classifications have been proposed for lymphangioma: (a) Landing and Farber classified LM into lymphangioma simplex, cavernous lymphangioma, and cystic lymphangioma/hygroma [5]; (b) based on the depth and size of abnormal lymphatic vessels, LM can be classified into superficial (lymphangioma circumscriptum) and deep (cavernous lymphangioma and cystic hygromas); (c) based on the onset, LM is divided into congenital (more common) and acquired; (d) based on the spread into anatomical spaces in the head and neck region [6], cystic hygroma can be divided into class/grade I - unilateral infrahyoid, class/grade II - unilateral suprahyoid, class/grade III - unilateral infra and suprahyoid, class/grade IV - bilateral suprahyoid and class/grade V - bilateral infra and suprahyoid [7]; (e) recently, ISSVA classifies LM into common (cystic) LM (macrocystic LM, microcystic LM, and mixed cystic LM), generalized lymphatic anomaly (GLA), Gorham-Stout disease, channel type LM, and "acquired" progressive lymphatic anomaly [2].

Surgical treatment remains the gold standard. However, there are certain limitations, including chances of bleeding, high chances of recurrences, scarring, especially with large-sized lesions, and esthetic and functional impairment. Furthermore, surgical treatment is limited or non-existent in patients with systemic diseases [8]. Considering these limitations, other modalities that can be advocated are laser therapy, cryotherapy, electrocautery, sclerotherapy, intralesional steroids, and combination injections of steroids, bleomycin, and bevacizumab [9]. Here, we present three cases of diverse forms of lymphatic malformations treated with two different modalities of treatment.

## Case presentation

Case 1

**Figure 1:**
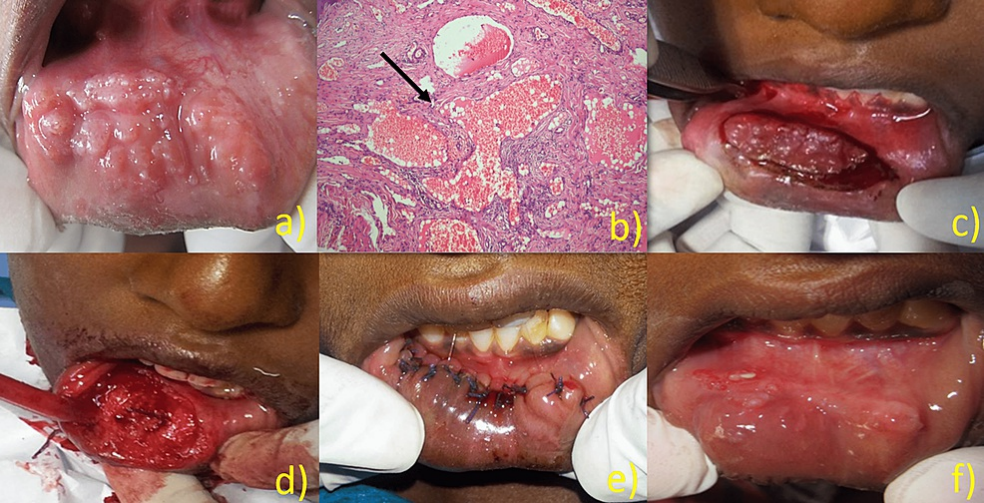
Case of lymphangioma of lower labial mucosa (a) Presence of multiple sessile polypoid growths in the lower labial mucosa; (b) histopathologically, it showed stratified squamous epithelium. Connective tissue demonstrated numerous dilated lymphatics containing lymph (c,d) intraoral operative procedure. (e) Post-operative view and (f) regression of the lesion with scarring.

A female patient aged 14 years reported to our department with a chief complaint of enlargement of the lower lip for four years. The patient gave a history of trauma in the same region for four years, after which the patient developed progressive enlargement of the lower lip. There was no relevant history of any pain, bleeding, or discharge. On inspection, the presence of diffuse enlargement of the lower lip with multiple sessile polypoid growths in the lower labial mucosa was evidenced, extending mediolaterally 1 cm medial from the right commissure of the lip to the left commissure. The mucosa over the growth had small pinpoint spiky projections (Figure 1a). The mucosa was normal in color with no visible pulsation. The growth was slightly tender and firm on palpation, with no pus discharge. The diascopy test was negative. A provisional diagnosis of lymphangioma and a differential diagnosis of cheilitis glandularis were made. Ultrasound revealed increased soft tissue swelling with multiple hypoechoic centers showing minimal vascularity. Histopathologically, it showed stratified squamous epithelium. Connective tissue demonstrated numerous dilated lymphatics containing lymph (Figure 1b). Based on clinical and histological examination, the final diagnosis of cavernous lymphangioma was made. Surgical excision was done under local anesthesia with the help of electrocautery (Figure 1c-1e). Postoperatively, the lesions healed with scarring (Figure 1f). There were small recurrences of the lesion in the right lateral region of the labial mucosa, which were removed in further sessions.

Case 2

**Figure 2:**
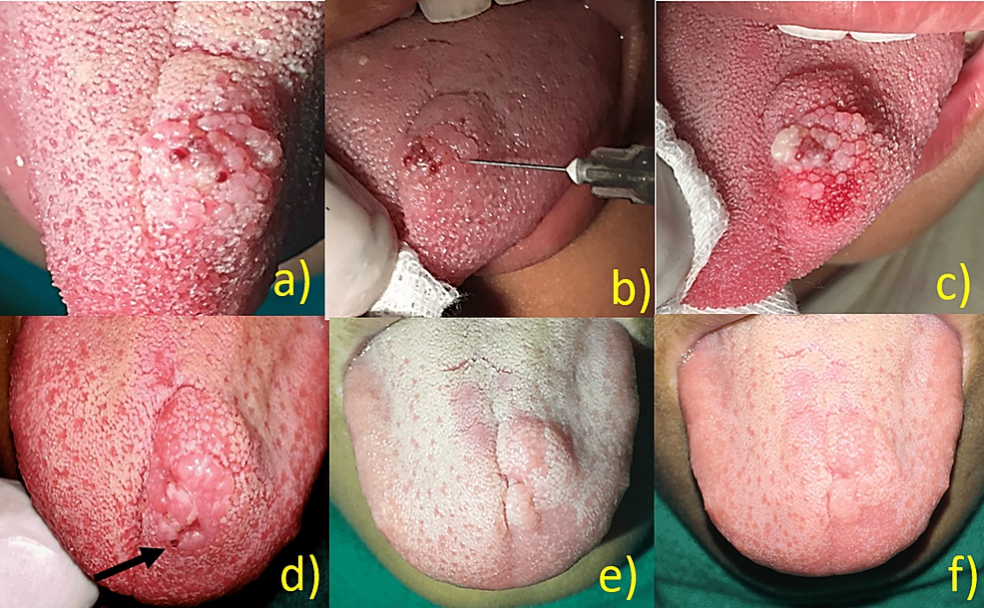
Case of lymphangioma of tongue (a) Presence of multiple pink to blue papules resembling a pebbly, vesicle-like surface; (b) intralesional 0.2 ml of sodium tetradecyl; (c) color change soon after injection; (d) ulceration is appreciated after three days; (e) 50% regression of the lesion; (f) complete regression of the lesion.

A female patient aged 12 years reported to our department with a chief complaint of enlargement of the dorsal surface of the tongue for three years. There was no history of any pain, bleeding, trauma, or discharge. The patient denied taking any medications. On intra-oral soft tissue examination, evidence of multiple polypoid growths on the left dorsal surface of the tongue was seen. Soft tissue examination revealed the presence of multiple pink to blue papules resembling a pebbly, vesicle-like surface - the so-called "frog-egg" or "tapioca pudding appearance." The mucosa over the growth had small, pinpointed, spiky projections. On palpation, no pulsation was felt. The mass was non-tender and firm, with no pus discharge (Figure 2a). The diascopy test was negative. A provisional diagnosis of lymphangioma circumscriptum was made. Ultrasound revealed increased soft tissue swelling with multiple hypoechoic centers showing minimum vascularity. It was managed conservatively with an intralesional injection of 0.2 ml of 3% sodium tetradecyl sulfate (Figure 2b). Mucosal blanching is appreciated soon after injection (Figure 2c). An ulcer developed two to three days after the injection (Figure 2d). In 20 days, a 50% reduction was observed (Figure 2e). Complete healing of the lesion was appreciated after 30 days without any scarring (Figure 2f). The patient is undergoing routine follow-up, and no recurrences have been observed to date.

Case 3

**Figure 3:**
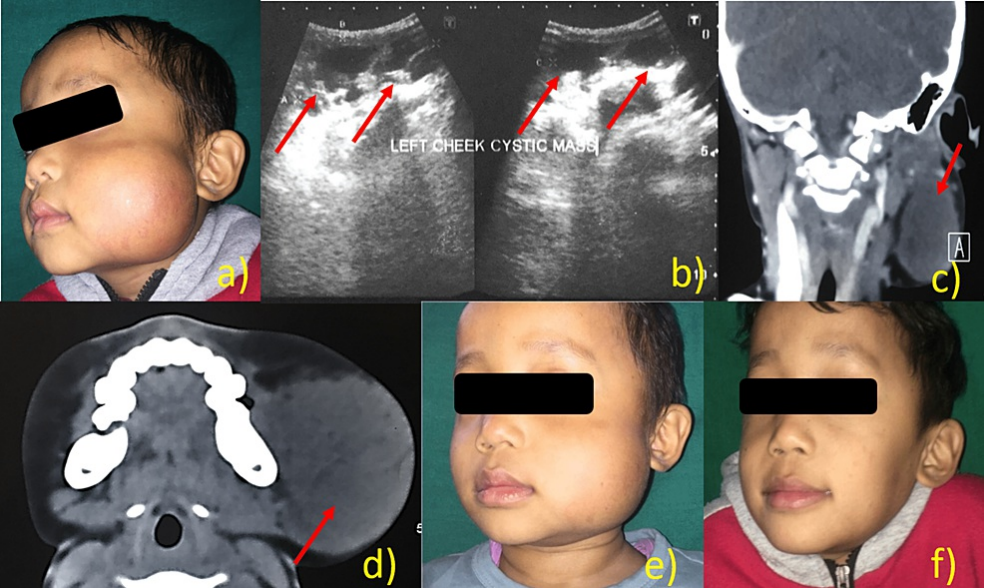
Case of lymphangioma/cystic hygroma (a) Dome-shaped swelling of the left face; (b) on ultrasonic revealed lobulated cystic lesion with multiseptation in the subcutaneous plane of the left cheek overlying masticator muscle; (c,d) coronal and axial CT revealed a well-defined, oval-shaped, thin-walled hypodense lesion of cystic attenuation with internal septation within subcutaneous planes of left maxillary regions; (e) 50% regression of the lesion; (f) complete regression of the lesion.

A male patient aged six years reported to our department with a chief complaint of swelling of the left face since childhood. The swelling was gradual in onset and gradually progressing in size. There was no relevant history of any tooth pain, bleeding, or discharge, and medication was appreciated. On extraoral examination, the presence of a dome-shaped swelling was seen, measuring about 6 cm × 6 cm in size. The skin over the swelling was stretched, shiny, and a little erythematous (Figure 3a). On palpation, the swelling was soft, fluctuant, nontender, and soft in consistency without any pulsatility. On ultrasonic examination, an approximately 6.3 cm × 2.6 cm × 5 cm lobulated cystic lesion with multiseptation was noted in the subcutaneous plane of the left cheek overlying the masticator muscle. No intrinsic vascularity was seen. The features were suggestive of lymphatic malformations (Figure 3b). On CT, there was a well-defined, oval-shaped, thin-walled hypodense lesion of cystic attenuation measuring 6.6 cm × 4.0 cm × 6.7 cm with internal septation within the subcutaneous planes of the left maxillary regions. On post-contrast images, there was a minimal enhancement of the lesional wall and internal septa (Figure 3c-3d). Based on clinical and radiological examination, the final diagnosis of lymphangioma/cystic hygroma was given. Fine needle aspiration cytology (FNAC) revealed scattered macrophages along with a few lymphocytes in a proteinaceous background. A bleomycin injection was planned after all necessary investigations were completed. After aspiration of lymphatic fluid, bleomycin injection was given intralesionally (0.5 mg/kg body weight, not exceeding 10 units at a time). The response was assessed by ultrasound. The patient was recalled after a month, and the procedure was repeated until complete remission. After the third injection, 50% regression was observed (Figure 3e), and complete regression was observed after the fifth injection (Figure 3f). The patient reported fever after each injection; thus, syrup paracetamol and syrup amoxicillin were prescribed. After a follow-up period of one year, no recurrences were observed.

## Discussion

Various theories have been suggested for the pathogenesis of lymphangiomas. As per the first theory, congenital LM can be the result of the sequestration of primary lymphatic channels and hence the inability to join main lymphatic vessels or veins. The second theory suggests that the lymphatic channels are unable to drain into the veins, thereby leading to lymphectasia and lymph stasis [10]. The latest theory suggests that the expression of vascular endothelial growth factor C (VEGF-C) and vascular endothelial growth factor receptor 3 (VEGF-R3) are raised in lymphangiomas, whereas the expression of pigment epithelium-derived factor and thrombospondin-1, which are two angiogenesis inhibitors, is lowered [11]. In some instances, acquired lymphangiomas can be the sequela of any disruption of previously normal lymphatic drainage, such as trauma, malignancy, surgery, and radiation therapy [4].

Clinically, superficial lymphangiomas or lymphangioma circumscriptum, appear as papillary/pebbly surfaces with clusters of translucent vesicles with the appearance of "frog-egg" or "tapioca pudding." It may be the same color as the surrounding mucosa or slightly reddish due to secondary hemorrhage. Cavernous lymphangiomas, on the other hand, appear as subcutaneous nodules, masses, or irregular nodularity of the surface with the normal mucosal color of grey or pink projection. Lastly, cystic hygroma, the most common type of lymphangioma, presents as a large, soft, circumscribed swelling with cyst-like cavities containing watery fluid. Cystic hygroma typically occurs on the neck, axilla, or groin. Intraorally, lymphangiomas affect the tongue (most commonly), labial mucosa, buccal mucosa, palate, and gingiva (very rarely) [3].

The diagnosis of lymphatic malformation/oral lymphangioma is clinical. Imaging modalities like color Doppler can help in identifying the condition. Ultrasonography can be used for the detection of the cystic nature and fluid component of the lesion, while angiography can be used to rule out vascular lesions. Biopsy remains the confirmative diagnosis [3].

Surgery is the preferred modality for the complete removal of lymphatic malformations. Surgical excision is mostly associated with recurrence if not removed completely. The recurrence rate varies from 10% to 53% [12]. Moreover, complications like damage to adjacent structures, scars, infections, respiratory obstructions, pneumonia, and palsies in the case of a large cystic hygroma warrant surgical excision. Thus, surgery is not advised in lymphatic malformations that are extensive, diffuse, and deep-seated. Even in our first case, we encountered recurrences and scarring [12].

Sclerotherapy involves the injection of a sclerosant that causes tissue irritation, endothelial damage, inflammation, and local tissue necrosis. Fibrosis and contracture eventually cause the lesion to disappear [7]. Because of their safety and efficacy, sodium tetradecyl sulfate (STS), OK-432, monoethanolamine oleate, polidocanol, hypertonic glucose solution, ethanol, sodium morrhuate, bleomycin, and corticosteroids have been used in various oral diseases, including vascular malformation, pyogenic granuloma, mucocele, and ranula [7,13,14].

Sodium tetradecyl sulfate or sodium l-isobutyl-4-ethyl octyl sulfate is a synthetically manufactured long-chain fatty acid, commonly used as a synthetic surfactant (soap), whereas bleomycin is an anti-tumor agent, which is a mixture of cytotoxic glycopeptide antibiotics isolated from a strain of Streptomyces verticillus. Bleomycin, through its non-specific inflammatory process, causes fibrosis and the resolution of the lesion [7,15,16].

Similar to our case, many studies have supported the use of sodium tetradecyl sulfate and bleomycin in the management of oral lymphangiomas [15,16]. Tiwari et al. [16] performed a study on 142 cases of lymphatic malformation with intralesional bleomycin injection. A complete response was noted in 80.3% of patients with the macrocystic variant, 67.4% with the microcystic variant, and 71.4% with the mixed type. The most common adverse effects were edema, erythema, and local induration with fever, similar to our third case.

## Conclusions

It is important to recognize these lesions early to achieve the best results and prevent recurrences. A multidisciplinary approach should be taken in the management of lymphatic malformations owing to their diverse presentations. Macrocystic lesions and lesions that are extensive warrant sclerotherapy as a better treatment modality compared to surgery. Finally, patients with any type of lymphatic malformation should be monitored on a regular basis because recurrence has been reported.

## References

[1] Syed NM (2016). Vascular lesions of head and neck: a literature review. Indian J Dent Sci.

[2] van Damme P, Kersloot MG, dos Santos Vieira B, Schultze Kool L, Cornet R (2022). The International Society for the Study of Vascular Anomalies (ISSVA) ontology. J Web Semantics.

[3] Shivhare P, Parihar A (2021). Benign and malignant non-odontogenic tumours of oral cavity. Textbook of Oral Medicine and Radiology.

[4] Eren S, Cebi AT, Isler SC, Kasapoglu MB, Aksakalli N, Kasapoglu C (2017). Cavernous lymphangioma of the tongue in an adult: a case report. J Istanb Univ Fac Dent.

[5] Kang HJ, Kim YH, Hwang YJ, Kim SY, Kim HS (2012). Upper extremity hemolymphangiomas in children: a case report. J Korean Soc Radiol.

[6] de Serres LM, Sie KC, Richardson MA (1995). Lymphatic malformations of the head and neck. A proposal for staging. Arch Otolaryngol Head Neck Surg.

[7] Mechery R, Kumar M, Arora P, Dinakar N (2016). Lymphangioma of lip treated with carbon dioxide laser. J Dent Lasers.

[8] Shivhare P, Singh V, Singh A (2019). Use of sodium tetradecyl sulphate for the treatment of oral lesions. JCMS Nepal.

[9] Hwang J, Lee YK, Burm JS (2017). Treatment of tongue lymphangioma with intralesional combination injection of steroid, bleomycin and bevacizumab. Arch Craniofac Surg.

[10] Hasan S, Ahmad SA, Kaur M, Panigrahi R, Panda S (2022). Lymphangioma of the lower lip-a diagnostic dilemma: report of a rare case with a brief literature review. Case Rep Dent.

[11] Yalçin M, Laçin N (2019). Lymphangioma in the buccal mucosa. J Craniofac Surg.

[12] Ognean ML, Boantă O (2012). Lymphangioma. Neonatology.

[13] Shivhare P, Haidry N, Sah N (2022). Comparative evaluation of efficacy and safety of the diode laser (980 nm) and sclerotherapy for the treatment of oral pyogenic granuloma. Int J Dent.

[14] Shivhare P, Haidry N, Sah N (2022). Comparative evaluation of efficacy and safety of the diode laser (980 nm) and sclerotherapy in the treatment of oral vascular malformations. Int J Vasc Med.

[15] Harjai MM, Jha M (2012). Intralesional bleomycin and sodium tetradecyl sulphate for haemangiomas and lymphangiomas. Afr J Paediatr Surg.

[16] Tiwari P, Pandey V, Bera RN, Sharma SP, Chauhan N (2021). Bleomycin sclerotherapy in lymphangiomas of the head and neck region: a prospective study. Int J Oral Maxillofac Surg.

